# Seroprevalence of toxoplasmosis among reproductive-aged women in Myanmar and evaluation of luciferase immunoprecipitation system assay

**DOI:** 10.1186/s12879-020-05650-y

**Published:** 2020-11-30

**Authors:** Khin Myo Aye, Eiji Nagayasu, Myat Htut Nyunt, Ni Ni Zaw, Kyaw Zin Thant, Myat Phone Kyaw, Haruhiko Maruyama

**Affiliations:** 1 Department of Medical Research, Ministry of Health and Sports, Yangon, Myanmar; 2 Division of Parasitology, Department of Infectious Diseases, Faculty of Medicine, University of Miyazaki, Kiyotake, Japan

**Keywords:** *Toxoplasma gondii*, Luciferase immunoprecipitation system, Nanoluciferase, Recombinant antigen, Enzyme-linked immunoassay, Myanmar

## Abstract

**Backgrounds:**

Primary infection with *Toxoplasma gondii* during pregnancy can pose serious health problems for the fetus. However, the epidemiological status of toxoplasmosis among reproductive-aged population in Myanmar is largely unknown. Although luciferase immunoprecipitation system (LIPS) assays for serodiagnosis of toxoplasmosis was developed mostly using mouse infection model, had not been tested by using field-derived human samples.

**Methods:**

A total of 251 serum samples were collected from reproductive-aged women, residing in Shwegyin township, Bago region, Myanmar and analyzed with a commercial ELISA kit, as well as in-house LIPS assays.

**Results:**

The overall seroprevalence for *Toxoplasma gondii* infection by the commercial ELISA was 11.5%. No clear risk factor was identified except for being in the younger age group (15–30 years old). Overall, LIPS assays showed low sensitivity when the commercial ELSA was used as a reference test.

**Conclusion:**

We identified the epidemiological situation of toxoplasmosis in some rural communities in Myanmar. The data obtained here will serve as a primary information for the effort to reduce toxoplasmosis in this region. Although looked promising in the previous experiments with mouse infection model, we found that the reported LIPS procedures need further improvements to increase the sensitivities.

## Background

Toxoplasmosis is a parasitic disease caused by *Toxoplasma gondii*. Between 30 and 50% of the global human population is thought to be infected by this protozoan pathogen [1]. Humans can be infected through accidental ingestion of oocysts excreted from infected cats, or by eating raw or undercooked meat containing tissue cysts [2]. Acute toxoplasmosis in most cases is asymptomatic and *T. gondii* can persist in host tissues by forming bradyzoites. In AIDS patients, *Toxoplasma* encephalitis is one of the most common brain lesions, which is lethal if left untreated [3]. Primary infection in pregnant women with *T. gondii* can lead to congenital toxoplasmosis [4]. Based on a recent meta-analysis, it was estimated that the transmission rates of *Toxoplasma* infection from acutely infected pregnant women to fetus are 5, 13 and 32%, if exposure was in 1st, 2nd or 3rd trimester, respectively [5]. If congenital transmission is confirmed, treatment with anti-parasitic drugs should be initiated to prevent complications of congenital toxoplasmosis. Therefore, diagnosing *Toxoplasma* infection early and precisely is of crucial importance.

Until now, serological tests have been the main diagnostic methods for screening of toxoplasmosis. Serological test based on Sabin-Feldman dye test has been considered the gold standard because of its high sensitivity and specificity in humans. However, it requires live parasites and highly skilled personnel and therefore is usually performed only in reference laboratories [6]. Other tests, such as indirect fluorescent antibody test (IFAT), latex agglutination test (LAT), indirect haemagglutination (IHA), modified agglutination test (MAT), and enzyme-linked immunosorbent assay (ELISA), are useful to screen *T. gondii* infection [7].

Luminescent assays are believed to be the most sensitive detection method due to the ability of signal multiplication and amplification [8]. Luciferase immunoprecipitation system (LIPS) is a relatively new luminescent assay technology, originally developed by Burbelo et al., that is based on liquid phase antibody binding event with luciferase-tagged antigens [9]. LIPS assay has been applied to a number of infectious diseases including strongyloidiasis [10], onchocerciasis [11], loiasis [12] and several autoimmune diseases such as type I diabetes, Sjogren’s syndrome, and systemic lupus erythematous in humans and demonstrated superior performances over conventional ELISA [13].

Recently, *T. gondii* recombinant fusion proteins of Nluc (a luciferase enzyme) and diagnostic antigens (rGRA6, rGRA7, rGRA8 and rBAG1) were produced and evaluated for use in LIPS assay. The newly developed LIPS protocols with these fusion recombinant antigens were tested using sera collected from mice experimentally infected with *T. gondii*, and an international standard anti-*Toxoplasma* human serum, TOXM [14].

The aim of this study is twofold: firstly, to estimate the sero-epidemiological status and risk factors in reproductive-aged women, living in this study areas of Myanmar by using a widely recommended commercial ELISA kits; and secondary to acquire information regarding the performance of the LIPS assays compared to the commercial ELISA kits.

## Methods

### Study population

**Fig. 1:**
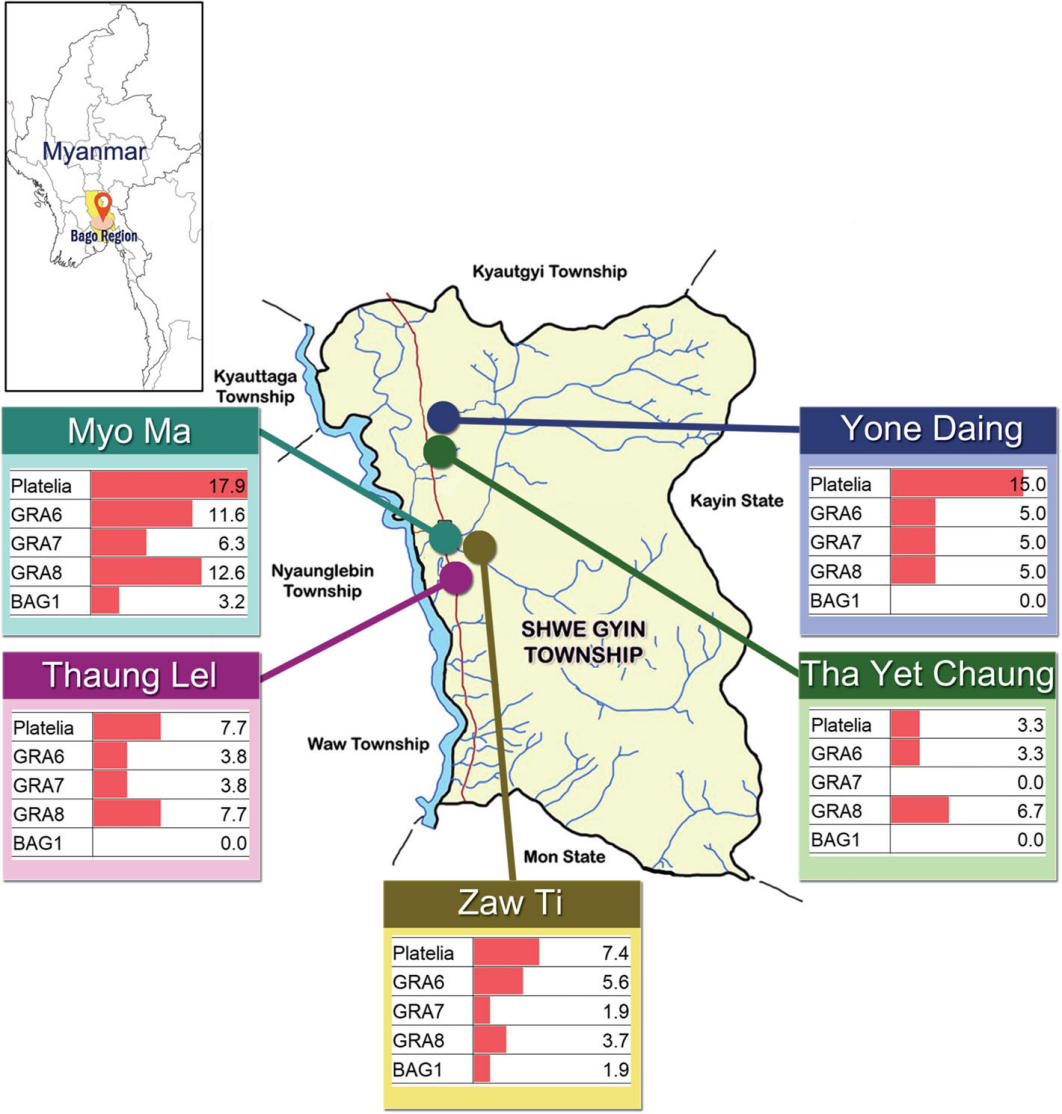
Sampling locations and seroprevalences calculated for each location; Thaung Lel, Zaw Ti, Tha Yet Chaung, Yone Daing and Myo Ma belong to Shwegyin Township, Bago Region, Myanmar. The seroprevalences based on Platelia IgG-ELISA and LIPS assays with Nluc-rGRA6, −rGRA7, −rGRA8 and –rBAG1 are presented

A cross-sectional descriptive study was carried out at five villages of the Shwegyin Township, Bago Region, Myanmar, from January to March, 2017. The minimal sample size required was estimated to be 246 with 5% precision and 95% confidence levels, assuming a prevalence of 20% [15]. Thereafter, adult women within reproductive age (15–55 years), apparently healthy were included in this study. Women who showed signs and symptoms of severe infection were excluded from this study. Villages in Shwegyin township (Fig. 1) were selected due to high population densities of cats and animals in their homes and surroundings. No documented data on seroprevalence of anti-*T. gondii* antibodies in female of reproductive age and associated risk factors were available at the time of the survey. Structured questionnaires (Table S1) were used to assess their knowledge and to explore the possible risk factors of toxoplasmosis in those population.

### Serum sample collection

Venous blood (1 mL) was collected under aseptic condition. The blood was allowed to clot for at least 30 mins at room temperature. Serum was separated by centrifugation at 1500 g for 15 mins and stored at − 20 °C until analyzed.

### Serological investigations

All the samples were measured for IgG antibodies against *T. gondii* by a commercial ELISA kit (Platelia™ Toxo-IgG, Bio-Rad, Marnes-la-Coquette, France; hereinafter called “Platelia IgG-ELISA”), according to the manufacture’s instruction. The positive samples by Platelia IgG-ELISA were tested again by Platelia™ Toxo-IgM (Bio-Rad, Marnes-la-Coquette, France). The same set of sera were tested with LIPS assays with four different recombinant *T. gondii* antigens (Nluc-rGRA6, −rGRA7, −rGRA8 and -rBAG1). For selected samples, additional testing was conducted by Architect Toxo-IgG (Abbott Laboratories, Abbott Park, IL, USA) at a clinical laboratory service provider, Rinsho Miyazaki, Japan.

The detailed procedures for LIPS assays can be found in our previous publication [14]. Briefly, the recombinant proteins were adjusted to the following concentrations by diluting the original stocks with buffer A (50 mM Tris pH 7.5, 100 mM NaCl, 5 mM MgCl2, 1% Triton X-100); Nluc-rGRA6 (0.4 ng/μL), Nluc-rGRA7 (1.5 ng/μL), Nluc-rGRA8 (1.0 ng/μL) and Nluc-rBAG1 (0.7 ng/μL). Sera were diluted 10-fold with buffer A as well. The diluted recombinant protein (50 μL), diluted serum (10 μL) and buffer A (50 μL) were mixed, and antibody binding to antigens were allowed to occur by incubating them at room temperature for 1 h. Protein A/G beads were added to capture the antigen-antibody complex. These immune complexes were washed eight times with 200 μL of buffer A and two times with 200 μL of PBS, then furimazine substrate (Nano-Glo luciferase assay substrate [Promega, Madison, WI, USA]) was added. Luciferase activity deriving from the Nluc-*T.gondii* antigen fusion proteins bound to the beads were measured by a luminometer (DTX 800 multimode detector, Beckman Coulter, Tokyo, Japan) and expressed as relative light unit (RLU). The serum samples, positive and negative controls were tested in duplicate wells for each run.

Seroreactivities to Nluc-rBAG1 antigen was further evaluated by conventional ELISA. Wells of ELISA plates (C96 Maxisorp Nunc-immunoplate; ThermoScientific, Roskilde, Denmark) were coated with 50 μL of coating buffer (50 mM carbonate/bicarbonate [pH 9.6]) containing 5 μg/mL of Nluc-rBAG1, overnight at 4 °C. By using a plate washer, AMW-8R (BioTec, Tokyo, Japan), the wells were washed with 300 μL of washing buffer (0.05% Tween 20-PBS) three times. The wells were then blocked with 150 μL of blocking buffer (1% [w/v] casein in TBS [Tris-buffered saline], pH 7.6) at room temperature for 2 h. After removing the blocking buffer, 50 μL of human sera diluted 1:100 with the blocking buffer were added and incubated at 37 °C for 1 h. The plates were washed with 300 μL of washing buffer three times, then incubated with 50 μL of secondary antibody (peroxidase-labeled polyclonal rabbit anti-human IgG, Dako, Glostrup, Denmark) at 1:2000 dilution in blocking buffer at 37 °C for 1 h. The wells were washed with 300 μL of washing buffer three times, then 50 μL of ABTS^R^ peroxidase substrate (1-component; KPL, Gaithersburg, MD, USA) was added into the each well, and kept at room temperature for 30 mins. The plates were measured at 405 nm by iMark™ microplate reader (Bio-Rad, Irvine, CA, USA). Each serum sample was run in duplicate wells. Positive and negative control were included in each run.

The same set of sera (i.e., 251 sera) were also tested with conventional ELISA using commercially available recombinant *T. gondii* P30 (SAG1) antigen (Bio-Rad, Irvine, CA, USA). All the procedures were the same as mentioned above except 2 μg/mL rSAG1 was used for coating each well.

### Statistical analysis

Statistical analyses were performed by using SPSS version 22.0 (IBM Corp., New York, NY, USA). For the categorical data, Chi-square test or Fisher’s exact test (when the minimum cell counts were less than 5) were used. Odds ratio (OR) with 95% confidence interval (CI) were also calculated. Sensitivity, specificity, positive predictive value, negative predictive value and Cohen’s Kappa value were calculated by SPSS. Correlation and Mann-Whitney t tests were used to compare antibody titers in different groups. Differences were considered significant if *P* < 0.05. Graphs were built using GraphPad Prism 7 (GraphPad Software Inc., San Diego, CA, USA).

## Results

### Demographic and behavioral profiles of the study population

The age of female included in this study ranged between 15 and 54 years (median age; 32 years). Among them, 72.5% (182/251) were married, 22.7% (57/251) had bad obstetric history and 64.1% (161/251) were employed. Regarding the contact with animals, 62.9% (158/251) owned a cat (s) and 53.8% (135/251) had stray cat (s) at their home respectively. In addition, 56.6% (142/251) answered other animals were present at their home or compounds. Total 71.3% (179/251) had a habit of eating raw vegetables after washing with water. Most of them (98.0% [246/251]) cooked meat thoroughly. Few people (10.0% [25/251]) live in their farms but 88.0% (221/251) had a history of contact with soil for gardening or cleaning their yards. Only 0.8% (2/251) had heard the word toxoplasmosis but most 72.5% (182/251) had awareness of disease transmission from cats through health workers, elder persons, friends, media such as radio, television and magazines.

### Seroprevalence and associated risk factors of *Toxoplasma* infection

Based on the results obtained by Platelia IgG-ELISA, the overall seroprevalence for *T. gondii* infection in our study population was calculated to be 11.5% (29/251), varying from 3.3 to 17.9% (Fig. 1).

The seroprevalence judged by the Plateria Toxo-IgG ELISA and associated factors calculated by bivariate analysis are shown in (Table 1). Being in the younger group was associated with increased risk of having positive test result (OR = 2.56; 95% CI = 1.14–5.88, *P* = 0.02). Contact with cats or presence of stray cats was not proven to be risk factors in this study group. The presence of other animals in their home or surroundings were shown to be negatively associated with positive antibody test results by Platelia IgG-ELISA (OR = 0.36; 95% CI = 0.16–0.81, *P* = 0.01).

**Table 1:** Bivariate analysis of selected demographic, environmental, behavioral variables with seroprevalence of *Toxoplasma gondii* infection based on Plateria IgG ELISA in (251 samples) reproductive-aged women

Variable	Number of positive residents (%)	Odds ratio (95% CI)	*p*
Age (years)			
15–30	19 (16.8)	2.56 (1.14–5.88)	0.02
> 30	10 (7.2)		
Occupation			
Employed	17 (10.6)		
Unemployed	12 (13.3)	1.3 (0.59–2.87)	0.51
Bad obstetric history			
No	14 (11.2)		
Yes	6 (10.5)	0.93 (0.34–2.57)	0.89
Contact with cat (s) or not			
No	6 (10.9)		
Yes	16 (15.5)	1.5 (0.55–4.09)	0.43
Presence of stray cat (s) at home			
No	15 (12.9)		
Yes	14 (10.4)	0.78 (0.36–1.69)	0.53
Presence of other animal (s)			
No	19 (17.4)		
Yes	10 (7.0)	0.36 (0.16–0.81)	0.01
Contact with other animal (s)			
No	4 (4.5)		
Yes	6 (11.1)	2.63 (0.71–9.77)	0.15
Drinking untreated water			
No	13 (15.7)		
Yes	16 (9.5)	0.57 (0.26–1.24)	0.16
Eating raw vegetables			
No	10 (13.9)		
Yes	19 (10.6)	0.74 (0.32–1.67)	0.46
Eating undercooked meat			
No	27 (11.0)		
Yes	2 (40.0)	5.41 (0.87–33.8)	0.07
Contact with soil			
No	3 (10.0)		
Yes	26 (11.8)	1.2 (0.34–4.24)	0.78

*P < 0.05 considered as significant. 95% CI = confidence interval

Groups that answered “No” were used as reference group for odds ratio calculation for yes/no questions. Groups of > 30 years old or employed were used as reference group for the odds ratio analysis for age and occupation, respectively

### Evaluation of LIPS assays

**Fig. 2:**
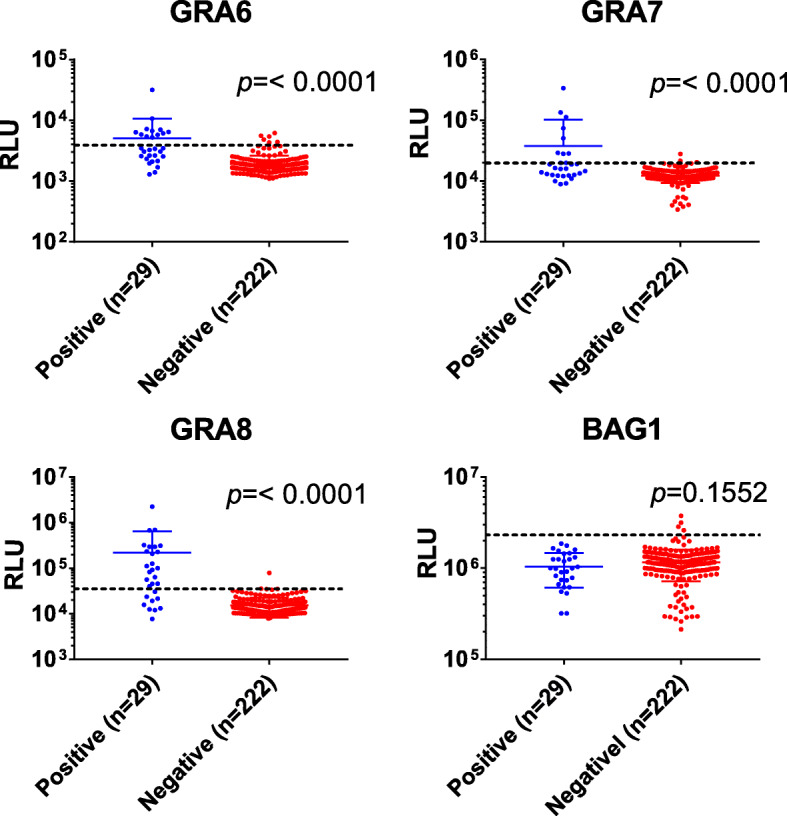
Detection of antibodies against Nluc-rGRA6, −GRA7, −GRA8 and -BAG1 by LIPS assays. Each dot represents mean RLU values of the duplicate wells for each serum sample. The blue dots (n = 29) and the red dots (n = 222) represent positive and negative samples determined by the Platelia IgG-ELISA, respectively. The dashed lines represent the cut-off values. Antibody titers in RLU (relative light unit) were plotted on log_10_ scale. Error bars with mean ± SD (standard deviation) are also shown over the scatter plots

LIPS assays were performed on the same set of sera. Because absolute cut-off values for LIPS assays have not been established, tentative cut-offs were calculated using the mean + 3SD RLUs obtained from all the negative samples by Platelia IgG-ELISA (*n* = 222). Based on these, 7.2% (18/251), 4.0% (10/251), 8.4% (21/251) and 1.6% (4/251) of the samples were judged as positive by LIPS assay with Nluc-rGRA6-, GRA7-, GRA8- and BAG1-LIPS assay, respectively. When Platelia IgG-ELISA was considered as a reference test, the sensitivities of LIPS assays were not high. In particular, none of the Platelia IgG-ELISA positive samples (*n* = 29) were positive with LIPS assay with Nluc-BAG1 (sensitivity = 0%) (Table S2). When RLU values were compared between Platelia IgG-ELISA positive and negative groups, the positive group had significantly higher RLU values than the negative group for Nluc-rGRA6-, GRA7- and GRA8-LIPS assays as expected. On the other hand, no statistically significant difference was observed for BAG1-LIPS assay (Fig. 2).

**Fig. 3:**
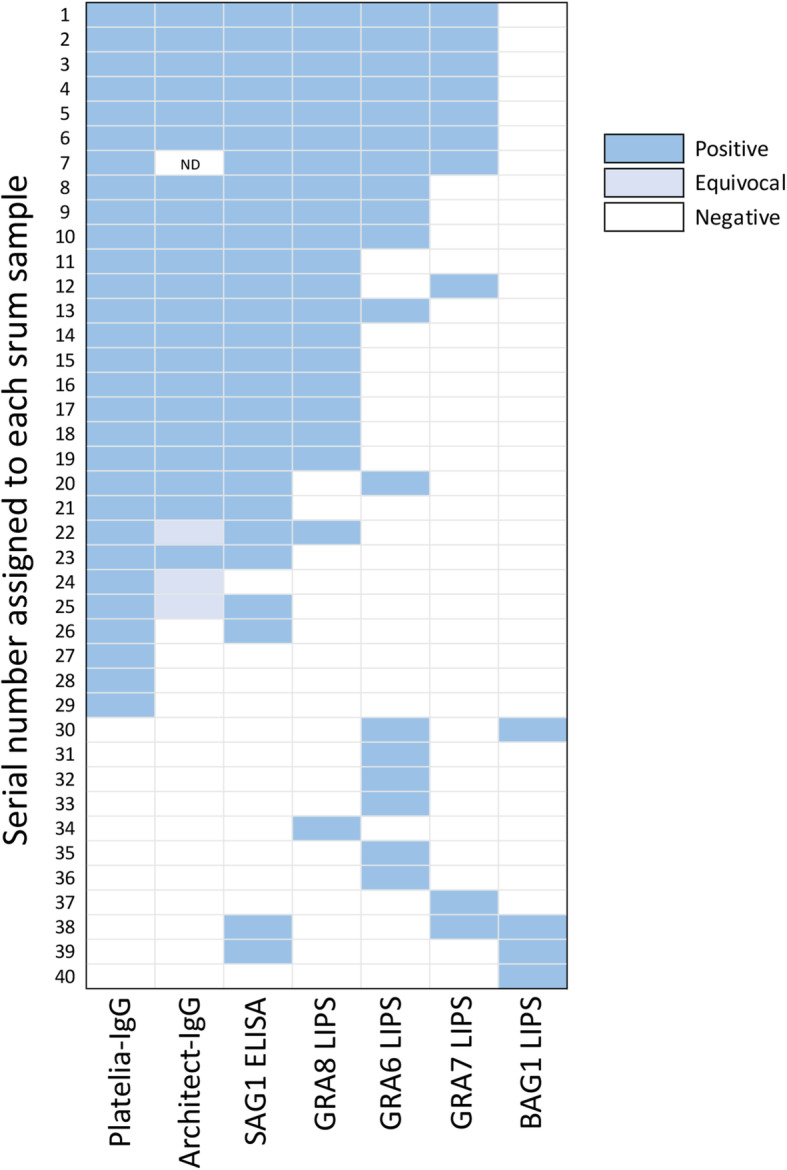
Pattern of seroreactivities against *T. gondii* antigens evaluated by Platelia IgG-ELISA, LIPS assays with four different *T. gondii* antigens and commercially available rSAG1 by conventional ELISA. Each positive test result is shown as a rectangle filled in blue, while negative test results are shown as blank areas. Numbers arranged along the Y-axis (identification [ID] numbers 1–40) represent sample identification numbers arbitrary assigned to each serum sample. For example, serum ID “No. 1” showed positive test results by all tests except for the BAG1-LIPS assay, while the serum ID “No. 40” was positive only by the GRA6-LIPS assay Samples that were diagnosed as negative by all tests (*n* = 211) are not shown in this figure

A total of 40 samples exhibited positive results by at least one of the six assays (Platetlia IgG-ELISA, LIPS assays with four different antigens and rSAG1-ELISA). The remaining samples (*n* = 211) were negative by all tests. The serological pattern of these 40 positive sera is illustrated in Fig. 3. Identification numbers were assigned to each of these 40 sera (numbers 1–40). Eleven sera (serum numbers from 30 to 40 in Fig. 3) were negative by Platelia IgG-ELISA but positive by LIPS assays with one or two recombinant antigens and by rSAG1-ELISA. None of the Nluc-rBAG1-LIPS positive sera were positive by Platelia IgG-ELISA (sample numbers 30, and 38–40).

**Fig. 4:**
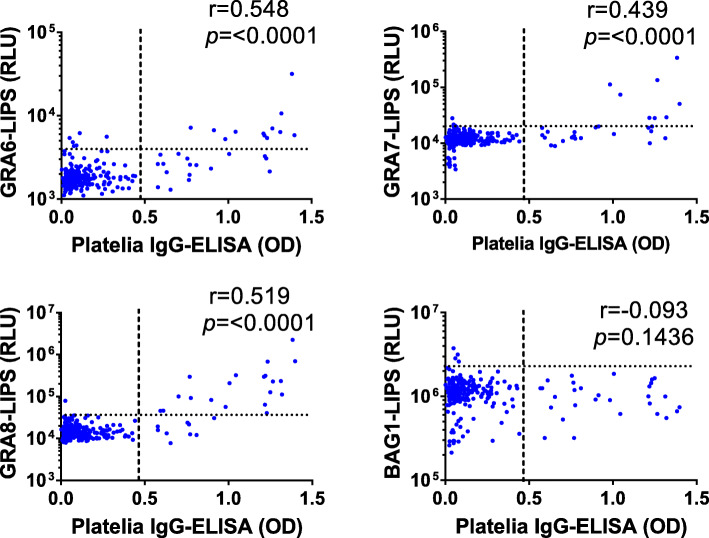
Correlation between Platelia IgG-ELISA and LIPS assays. The ELISA OD (optical density) values are plotted on the x-axis, while the LIPS RLU values are plotted on the y-axis (in log10 scale). Each dot indicates mean values of duplicate wells for each sample. The dashed lines represent cut-off values. There is a moderate correlation between Platelia IgG-ELISA and Nluc-rGRA6-LIPS assay (r = 0.548, *P* < 0.0001), rGRA8-LIPS assay (r = 0.519, *P* < 0.0001); fair correlation with rGRA7-LIPS assay (r = 0.439, *P* < 0.0001) respectively and statistically significant whereas there is no correlation between Platelia IgG-ELISA and Nluc-rBAG1-LIPS assay and statistically not significant (r = − 0.093, *P* = 0.1436)

Correlations between OD values obtained from Platelia IgG-ELISA and RLU values from LIPS assay were assessed. Moderate positive correlations were found in case of Platelia IgG-ELISA and GRA6-, GRA7- and GRA8-LIPS assays (Fig. 4). On the other hand, no correlation was found between Platelia IgG-ELISA and BAG1-LIPS assay. The RLU values obtained from Nluc-rBAG1-LIPS and OD values obtained from Nluc-rBAG1-ELISA showed a moderate correlation (r = 0.577, *P* < 0.0001) (Fig. S1).

**Fig. 5:**
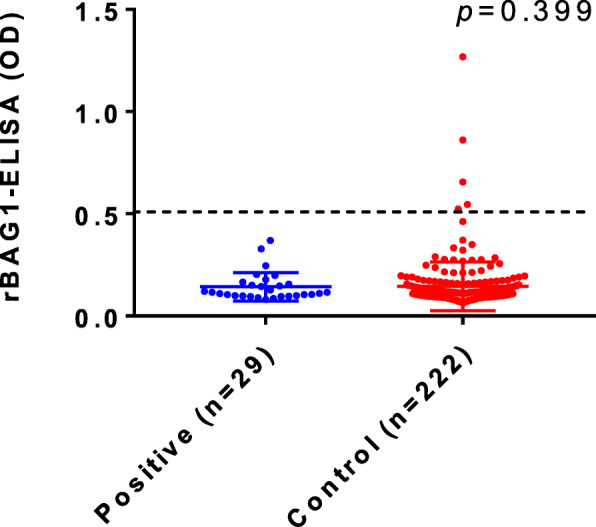
Detection of IgG antibodies against Nluc-rBAG1 by conventional ELISA. Each dot represents mean values of the duplicate wells for each serum sample. The blue dots (*n* = 29) and the red dots (*n* = 222) represent positive and negative samples determined by Platelia IgG-ELISA, respectively. Antibody titers in OD (optical density) are plotted on the y-axis

Similarly to the results obtained by Nluc-rBAG1-LIPS (Fig. 2), none of the Platelia IgG-ELISA positive sera were judged as positive by Nluc-rBAG1-ELISA. Figure 5 shows the results of ELISA with rBAG1. There were five samples judged positive in this assay (five dots located above the cut-off line). All of these five positive samples by this rBAG1-ELISA belonged to the group of Platelia IgG-ELISA negative individuals (Fig. 5, shown as Control [*n* = 222] in the figure).

All serum samples were also evaluated with rSAG1-ELISA (Platelia IgG-ELISA was considered as a reference test) and the seroprevalence was (27/251) 10.7% in which two samples were also positive by Nluc-rBAG1-LIPS assay and rBAG1-ELISA.

Commercial Platelia IgG-ELISA positive sera and LIPS assay positive (ie. Platelia IgG-ELISA negative) were also assessed by Platelia™ Toxo-IgM. Only one sample was positive and one was equivocal by Platelia™ Toxo-IgM respectively.

Given some degree of difference between test results obtained from Plateria IgG-ELSIA and LIPS assays, a subset of samples were also tested with Architect IgG ELSIA. All the Plateria IgG-positive samples except four were diagnosed either as positive or equivocal by Architect IgG ELSIA (Fig. 3). All the randomly selected Plateria IgG ELISA negative samples (*n* = 20) were also negative by Architect IgG ELISA. The resultant kappa value was 0.260, which was interpreted as fair agreement.

## Discussion

Primary *T. gondii* infection in pregnant women can lead to several adverse outcomes such as miscarriage and fetal damage. In this study, 251 sera obtained from healthy reproductive-age women living in rural area of Myanmar were examined. The prevalence of toxoplasmosis based on Platelia IgG-ELISA was 11.5% (29/251), which was lower than the seroprevalence reported previously in Myanmar, such as 23.5% among school children in Pyin Oo Lwin and Naung Cho [16], 31.7% among refugee/migrant pregnant women from Myanmar [17] and 30.7% among pregnant women attending at the antenatal clinic at Yangon Central Hospital [15].

The younger (15–30 years old) was associated with higher chance of presenting Platelia IgG-ELISA positive test results. In general, improvement of overall sanitary conditions is expected to decrease the seropositivities in younger generations. However, such trend was not observed in this study. Counterintuitively, presence of other animal (s) was found to be negatively associated with the seropositivity. This may not be a direct association but rather a consequence of confounding, necessitating more investigation. Contact with cat is thought to be one of the risk factors contracting toxoplasmosis [18]. In this study, more percentage of the residents with contact with cats had positive Plateria IgG-ELISA results. However, the difference could not be proven to be statistically significant, which may due to the small sample size.

Using the same set of the sera, LIPS assays with four different recombinant antigens were assessed. Various degrees of discordance among tests were observed by LIPS assays with four different recombinant *T. gondii* antigens. While specificities and negative predictive values were high, sensitivities were low overall. Among LIPS assays with the four antigens, the assay with Nluc-rGRA8 exhibited the highest agreement with the Plateria IgG-ELISA test results. Even with that test, the sensitivity was only 68.97%. Therefore, although initially looked promising based on the mouse experimental infection model, application of the reported LIPS protocol to human clinical samples require further improvements.

## Conclusions

The overall seroprevalence for *Toxoplasma gondii* infection by the Plateria IgG-ELISA was 11.5% in this study population in reproductive-aged women, based on the Plateria IgG-ELISA. This data will serve as a primary information for the effort to reduce toxoplasmosis in this region. The current LIPS assay protocols with recombinant *T. gondii* antigens need improvements to increase their sensitivities.

## Supplementary Information

**Additional file 1: Table S1**. Seroprevalence of toxoplasmosis among reproductive-aged women in Myanmar measured by luciferase immunoprecipitation system assay.

**Additional file 2: Table S2**. Comparison of diagnostic performances of (inactivated *T.gondii* antigen) commercial ELISA kit (Platelia IgG-ELISA) and (Nluc-rGRA6, −rGRA7, −rGRA8 and -rBAG1) LIPS assay.

**Additional file 3: Figure S1**. Correlation between the seroreactivities against Nluc-rBAG1 evaluated by ELISA and LIPS assays. The OD (optical density) values obtained by ELISA and the RLU (relative light unit) values obtained by LIPS are plotted on the x- and the y-axis, respectively. Each dot represents mean value of duplicate wells for each sample. There is a good correlation (r = 0.577) and statistically significant (*P* < 0.0001) between the two assays.

## Data Availability

The datasets used and/or analysed during the current study available from the corresponding author on reasonable request.

## Abbreviations

CI: Confidence interval
ELISA: Enzyme-linked immunosorbent assay
IgG: Immunoglobulin G
LIPS: Luciferase immunoprecipitation system
OD: Optical density
OR: Odds ratio
RLU: Relative light unit
