# Supplementary material for: Seroprevalence of toxoplasmosis among reproductive-aged women in Myanmar and evaluation of luciferase immunoprecipitation system assay
Source: BMC Infect Dis. 2020 Nov 30;20:906. doi: 10.1186/s12879-020-05650-y (PMC7706230; doi:10.1186/s12879-020-05650-y)
Supplement: Supplementary file 1 — Additional file 1: Table S1. Seroprevalence of toxoplasmosis among reproductive-aged women in Myanmar measured by luciferase immunoprecipitation system assay. [file 12879_2020_5650_MOESM1_ESM.docx]

Table S1

**Seroprevalence of toxoplasmosis among reproductive-aged women in Myanmar measured by luciferase immunoprecipitation system assay**

| **Sr** | **Description** | **Option** | **Code** |
| --- | --- | --- | --- |
| 1 | Date |  |  |
| 2 | Code Number |  |  |
| 3 | Age in years |  |  |
| 4 | Occupation | 1) Agricultural worker  2) Rubber plantation worker  3) Manual worker  4) Student  5) Dependent  6) Others |  |
| 5 | Education | 1) Illiterate  2) Primary school level  3) Middle and high school level  4) Graduate |  |
| 6 | Marital status | 1) Married  2) Unmarried |  |
|  | **RISK EXPOSURED** |  |  |
| 7 | Bad obstetric history | 1) Abortion  2) Still birth  3) Congenital abnormalities  4) Die within 1 year after born  (Mention if know the causes….)  5) Die > 1 year after born  (Mention if know the causes….)  6) Others |  |
| 8 | Presence of own cat in home | 1) Yes (number……………)  2) No |  |
| 9 | Contact history with own cat | 1) Yes  2) No  3) Not Relevant |  |
| 10 | Presence of stray cat(s) in home | 1) Yes  2) No |  |
| 11 | History of Cleaning litter tray | 1) Yes  2) No  3) Not Relevant |  |
| 12 | Frequency of changing cleaning litter tray | 1) Daily  2) 2-3 times per week  3) Infrequently  4) Never |  |
| 13 | Presence of other animals at home/from Surroundings | 1) Yes  2) No |  |
| 14 | Contact with that animals | 1) Yes  2) No  9) Not Relevant |  |
| 15 | Contact with cat/animals feces/urine | 1) Yes  2) No  9) Not Relevant |  |
|  | **HABITS AND BEHAVIORS** |  |  |
| 16 | Drinking untreated water | 1) Yes  2) No |  |
| 17 | Drinking sources of water | 1) Shallow well  2) Deep well  3) Purified well  4) Stream water |  |
| 18 | Drinking unpasteurized milk | 1) Yes  2) No |  |
| 19 | Consumption of raw vegetables after washing | 1) Yes  2) No |  |
| 20 | Consumption of undercooked meat | 1) Yes  2) No |  |
| 21 | Working in the garden/home with your hands in the soil | 1) Yes  2) No |  |
| 22 | Living in the Farm | 1) Yes  2) No |  |
| 23 | Habit of hand washing before eating | 1) Yes  2) No |  |
| 24 | If yes, | 1) Only with water  2) Always with soap and water  3) Occasionally with soap and  water  9) Not Relevant |  |
| 25 | Habit of hand washing after contact with soils | 1) Yes  2) No |  |
| 26 | If yes, | 1) Only with water  2) Always with soap and water  3) Occasionally with soap and  water  9) Not Relevant |  |
|  | **ANTIBIOTICS AND BLOOD TRANSFUSION** |  |  |
| 27 | History taking antibiotics within one month | 1) Yes  2) No |  |
| 28 | If Yes, | 1)Within 1 month  2) More than 1 month |  |
| 29 | Drugs name | 1) Yes (Mention …………….)  2) No  3) Don’t know |  |
| 30 | History blood transfusion | 1) Yes  2) No |  |
| 31 | If Yes, | 1) Within 1 year  2) More than 1 year  3) Not remember  9) Not Relevant |  |
|  | **AWARENESS OF TOXOPLASMOSIS** |  |  |
| 32 | Have you heard about Toxoplasmosis? | 1) Yes  2) No |  |
| 33 | Do you know disease transmission from cats or not? | 1) Yes  2) No |  |
| 33 | If yes, from whom did you hear? | 1) Health worker  2) Elder persons  3) Friends  4) Media (TV, Radio, Books,  Magazines, etc)  5) Others |  |
|  | **Results of *Toxoplasma* Antibody** |  |  |
| 34 | Results of *Toxoplasma* Antibody | IgG, IgM test |  |
