# Supplementary material for: Seroprevalence of toxoplasmosis among reproductive-aged women in Myanmar and evaluation of luciferase immunoprecipitation system assay
Source: BMC Infect Dis. 2020 Nov 30;20:906. doi: 10.1186/s12879-020-05650-y (PMC7706230; doi:10.1186/s12879-020-05650-y)
Supplement: Supplementary file 2 — Additional file 2: Table S2. Comparison of diagnostic performances of (inactivated T.gondii antigen) commercial ELISA kit (Platelia IgG-ELISA) and (Nluc-rGRA6, −rGRA7, −rGRA8 and -rBAG1) LIPS assay. [file 12879_2020_5650_MOESM2_ESM.docx]

**Table S2**

Comparison of diagnostic performances of (inactivated *T.gondii* antigen) commercial ELISA kit (Platelia IgG-ELISA) and (Nluc-rGRA6, -rGRA7, -rGRA8 and -rBAG1) LIPS assay

| LIPS assay | Sen | Spec | PPV | NPV | Kappa |
| --- | --- | --- | --- | --- | --- |
|  | (%) | (%) | (%) | (%) |  |
| Nluc-rGRA6 | 41.38 | 97.3 | 66.67 | 92.7 | 0.18 |
| 95% CI | (23.52-61.06) | (94.21-99.00) | (44.84-83.11) | (90.34-94.52) | (0.09–0.27) |
|  |  |  |  |  |  |
| Nluc-rGRA7 | 27.59 | 99.1 | 80 | 91.29 | 0.22 |
| 95% CI | (12.73-47.24) | (96.78-99.89) | (47.15-94.72) | (89.32-92.92) | (0.13–0.31) |
|  |  |  |  |  |  |
| Nluc-rGRA8 | 68.97 | 99.55 | 95.24 | 96.09 | 0.37 |
| 95% CI | (49.17-84.72) | (97.52-99.99) | (73.59-99.31) | (93.45-97.69) | (0.31–0.43) |
|  |  |  |  |  |  |
| Nluc-rBAG1 | 0 | 98.2 | 0 | 88.26 | -0.01 |
| 95% CI | (0.00-11.94) | (95.45-99.51) |  | (88.07-88.44) | (-0.03–0.00) |

Abbreviations:

CI: confidence interval, Sen: sensitivity, Spec: specificity, PPV: positive predictive value, NPV: negative predictive value, Nluc: nanoluciferase, rGRA6: recombinant GRA6, rGRA7: recombinant GRA7, rGRA8: recombinant GRA8, rBAG1: recombinant BAG1. The cut-off values are established as mean+3SD (standard deviation) of values obtained from all the negative samples (ie. n=222) by Platelia IgG-ELISA (as a reference test).
