# Supplementary figures and images for: Seroprevalence of toxoplasmosis among reproductive-aged women in Myanmar and evaluation of luciferase immunoprecipitation system assay
Source: BMC Infect Dis. 2020 Nov 30;20:906. doi: 10.1186/s12879-020-05650-y (PMC7706230; doi:10.1186/s12879-020-05650-y)

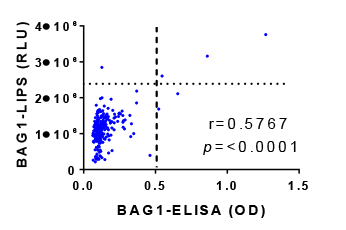

Supplement: Supplementary file 3 — Additional file 3: Figure S1. Correlation between the seroreactivities against Nluc-rBAG1 evaluated by ELISA and LIPS assays. The OD (optical density) values obtained by ELISA and the RLU (relative light unit) values obtained by LIPS are plotted on the x- and the y-axis, respectively. Each dot represents mean value of duplicate wells for each sample. There is a good correlation (r = 0.577) and statistically significant (P < 0.0001) between the two assays. [file 12879_2020_5650_MOESM3_ESM.tif]
